# The effect of the stress hormone cortisol on the metatranscriptome of the oral microbiome

**DOI:** 10.1038/s41522-018-0068-z

**Published:** 2018-10-18

**Authors:** Ana E. Duran-Pinedo, Jose Solbiati, Jorge Frias-Lopez

**Affiliations:** Department of Oral Biology, University of Florida, College of Dentistry, 1395 Center Drive, Gainesville, FL 32610-0424 USA

## Abstract

Imbalances of the microbiome, also referred to as microbial dysbiosis, could lead to a series of different diseases. One factor that has been shown to lead to dysbiosis of the microbiome is exposure to psychological stressors. Throughout evolution microorganisms of the human microbiome have developed systems for sensing host-associated signals such as hormones associated with those stressors, enabling them to recognize essential changes in their environment, thus changing their expression gene profile to fit the needs of the new environment. The most widely accepted theory explaining the ability of hormones to affect the outcome of an infection involves the suppression of the immune system. Commensal microbiota is involved in stressor-induced immunomodulation, but other biological effects are not yet known. Here we present the impact that cortisol had on the community-wide transcriptome of the oral community. We used a metatranscriptomic approach to obtain first insights into the metabolic changes induced by this stress hormone as well as which members of the oral microbiome respond to the presence of cortisol in the environment. Our findings show that the stress hormone cortisol directly induces shifts in the gene expression profiles of the oral microbiome that reproduce results found in the profiles of expression of periodontal disease and its progression.

## Introduction

In recent years a considerable effort has been placed on characterizing the different microbial communities colonizing the human body.^1^ However, the nature of host-microbial interactions in the microbiome that allow for the maintenance of a stable microbiota is still poorly understood. Among the environmental factors that may alter the equilibrium in host-microbiome homeostasis, host-stress is a known risk factor for a variety of diseases. In case of acute stress, stress response may prepare the immune system for challenges such as infection, but when it becomes chronic, it may influence inflammatory processes leading to the development of systemic or local diseases such as rheumatoid arthritis,^2^ diabetes,^3^ or periodontitis.^4^ Furthermore, physiological stress can also alter the composition of the commensal microbiota in the human microbiome.^5^

The most widely accepted theory to explain how hormones can influence microbial infections involves the of the immune system. According to this model, stress can activate the central nervous system and the hypothalamus releases corticotropin-releasing hormone and arginine vasopressin that stimulates the release of adrenocorticotropin from the pituitary, which in turn results in the production of cortisol by the adrenal cortex. However, almost immediately following its first use, cases of adrenaline-associated sepsis were reported.^6^ It was demonstrated that the dose of *Clostridium* needed to cause infection was significantly smaller when was injected in the presence of a therapeutic level of adrenaline.^7^ Since then, there have been reports associating the levels of neuroendocrine hormones, such as adrenaline, with infectious diseases, suggesting organisms themselves directly respond to the presence of stress hormones. The study of these interactions has been termed ‘microbial endocrinology’.^8,9^ Microorganisms that have evolved systems for sensing host-associated signals such as hormones would have an evolutionary advantage over those that have not. Detecting such signals enables the microbiome to recognize essential changes in its environment thus changing its expression gene profile to fit the needs of the new environment. Moreover, there is also the possibility that the microbiome not only responds to human hormones but may be responsible for controlling their levels. Just recently it has been proposed that *Ruminococcus*, a member of the gut microbiome in piglets, controls levels of n-acetylaspartate, the second most abundant molecule in the brain, via levels of cortisol.^10^

Although most investigations of stress hormones induction of growth and virulence have been carried out with gut-associated bacteria, a few studies have shown that stress hormones have a significant effect on the growth of periodontal pathogens.^11,12^ In the oral cavity glucocorticoids, including cortisol, depress immunity by inhibiting the production of secretory immunoglobulins, and neutrophil functions, all of which may impair defense against infection by periodontal microorganisms.^13^ Cortisol or hydrocortisone is the primary hormone responsible for the stress response, and its levels increase in saliva and serum with the severity of periodontal disease.^14,15^ We hypothesize that the oral microbiome is capable of sensing changes in the levels of stress hormones and its response could be associated with severity of periodontal disease. Here we present the results of an in vitro study to assess the effect that cortisol had on the community-wide transcriptome of the oral community. We used a metatranscriptomic approach to obtain first insights into the metabolic changes induced by this stress hormone as well as which members of the oral microbiome respond to the presence of cortisol in the environment.

## Results and discussion

**Fig. 1:**
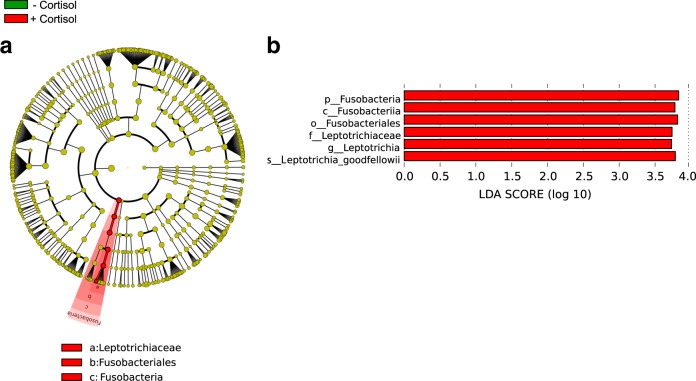
Distinct active taxa identified using LEfSe analysis. Metatranscriptome hit counts were obtained using Kraken against an oral microbiome database. Counts were then analyzed using LEfSe to identify significant differences at the species level between the microbial communities compared. **a** Cladogram constructed using the LEfSe method to indicate the phylogenetic distribution of active bacteria that were remarkably enriched. Red represented the enriched taxa the untreated microbial community and green represented the enriched taxa in the microbial community treated with cortisol. **b** LDA (Linear Discriminant Analysis) scores showed significant bacterial differences within groups at different taxonomic levels. Red represented the enriched taxa in the untreated microbial community and green represented the enriched taxa in the microbial community treated with cortisol

Our first experiments consisted of treating samples of dental plaque with cortisol at a concentration found in the saliva of patients with periodontitis.^14,16^ As a control for these experiments we used dental plaque that was incubated under the same conditions as the treatment samples but without cortisol added to the saliva used as the medium. People with periodontal disease present higher levels of cortisol in the gingival crevicular fluid,^14^ a serum exudate in direct contact with the oral microbiome. After only 2 h of incubation in the presence of cortisol, we proceed to perform the analysis, avoiding possible changes related to the growth of individual members of the microbiome and not to the presence of the hormone itself (see Methods section in the Supplementary Information accompanying this manuscript). We assigned the phylogenetic origin of those sequences using Kraken,^17^ and phylogenetic profiles were used to identify significant differences between active communities under the different conditions studied by performing linear discriminant analysis (LDA) effect size (LEfSe). In our study, LEfSe determines the active members of the oral microbiome, based on changes in the number of transcripts, that most likely explain differences between the two biological conditions tested (presence and absence of cortisol). LEfSe robustly identifies features that are statistically different among biological classes using the non-parametric factorial Kruskal-Wallis (KW) sum-rank test and subsequently uses a set of pairwise tests among subclasses using the (unpaired) Wilcoxon rank-sum test for biological consistency. Finally, LDA estimates the effect size of each differentially abundant taxonomic group.^18^ Among all the organisms in the oral community, members of the phylum Fusobacteria (class Fusobacteriia and order Fusobacteriales) were significantly more active (increased the number of transcripts significantly) after the addition of cortisol (Fig. 1). Among them, one species, *Leptotrichia goodfellowii*, was substantially more active (Fig. 1b). Species belonging to the *Fusobacteriales*, such as *Fusobacterium nucleatum* have been associated with a wide variety of human diseases, other than periodontitis, including adverse pregnancy outcome, GI disorders (e.g., colorectal cancer, inflammatory bowel disease), cardiovascular disease, rheumatoid arthritis, and respiratory tract infections.^19^ *Leptotrichia* species are typically part of the commensal flora in the oral cavity and genitourinary tract and are seldom found in clinically significant specimens. However, *Leptotrichia* has been found to be in higher proportion in gingivitis.^20,21^

**Fig. 2:**
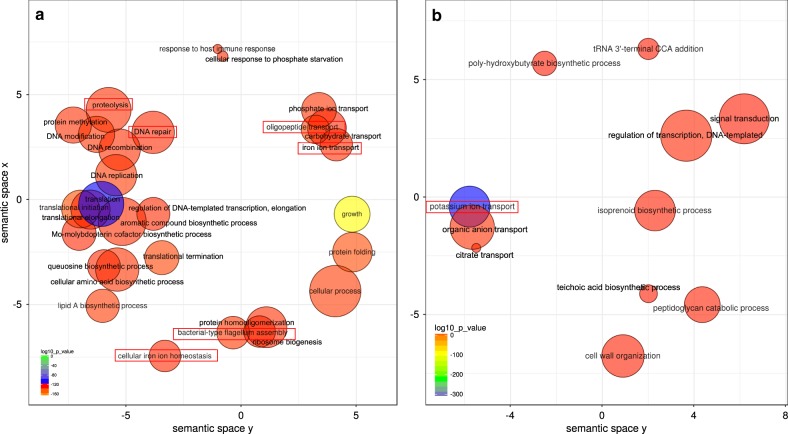
Changes in functional profiles in the oral microbiome treated with cortisol. We used Gene Ontology (GO) enrichment analysis to assess the oral microbiome functional response to the presence of added cortisol to the medium. Enriched terms obtained using GOseq were summarized and visualized as a scatter plot using REVIGO. Only GO terms with FDR adjusted *p*-value < 0.05 in the ‘GOseq’ analysis were used. **a** Over-represented functional activities in the presence of cortisol summarized as GO terms related to biological processes. **b** Over-represented functional activities in the absence of cortisol summarized as GO terms related to biological processes. Circle size is proportional to the frequency of the GO terms; color indicates the log10 *p*-value (red higher, blue lower). The distance between circles represents GO terms’ semantic similarities. Each of the circles represents a GO term, which depending on the similarity in the terms included in them they will be closer or more distant in the graph. In red are activities we have previously seen associated with periodontitis and its progression.^22,23^

Next, we looked at how cortisol influenced the profiles of expression of the oral microbiome. To this end, we performed enrichment of Gene Ontology (GO) terms analysis, and we observed that, after only 2 h of exposure to the hormone, the profiles of activities of the whole community had already changed and they were similar to the profiles of expression we previously found in periodontitis progression.^22,23^ Among them GO terms associated with proteolysis, oligopeptide transport, iron metabolism, and flagellum assembly were over-represented in the presence of cortisol (Fig. 2a). What is more important these activities have been associated with an increase in pocket depth, and essential clinical parameter in periodontitis, during the progression of the disease.^23^ Interestingly, GO terms linked to response to host immune response were also over-represented when cortisol was present, even though the microbiome in our samples was not in contact with host cells (Fig. 2a). As previously indicated potassium ion transport was significantly under-represented when cortisol was added (Fig. 2b), mimicking our in vivo observations of periodontitis progression.^23^ In a follow-up manuscript, we demonstrated that ion potassium is a signal in functional dysbiosis of periodontal disease.^24^

Interestingly, when we look at the activities associated explicitly with putative virulence factors, we found a similar pattern of over-represented GO terms related to the addition of cortisol (Sup. Figure 1). Although phylum Fusobacteria was the phylogenetic group that increased its transcriptional activity more significantly, the rest of the microbiome showed a shift in its profile of expression. Thus, the more significant fraction of up-regulated putative virulence factors seems to be synthesized by members of the genus *Streptococcus* (Sup. Figure 2). We observed similar results in our two previous studies on periodontitis progression^23^ and the effect of ion potassium in functional dysbiosis of the oral microbiome.^24^ As a whole, these results seem to indicate that the presence of cortisol leads to a community-wide response very similar to the one observed in vivo during periodontitis.

We then used a simpler system based on our previous results on the community-wide transcriptome results. To this end, we performed transcriptome analysis of the effect of cortisol on pure culture of organisms that we identified as more active in the presence of cortisol (Fig. 1): *Leptotrichia goodfellowii* and *Fusobacterium nucleatum*, which is a representative of the order Fusobacteriales and an essential member of the oral microbiome. In just 2 h of exposure, there was a shift in their transcriptome profiles. *F. nucleatum* showed an increase in biological processes GO terms associated with proteolysis, cobalamin biosynthesis and iron transport (Sup. Fig. 3a), which we have previously found associated with the progression of periodontal disease.^22,23^ In the case of *L. goodfellowii*, we also found activities related to iron ion transport (Sup. Fig. 3b). The intersection of metabolic events that were commonly altered in *F. nucleatum* and *L. goodfellowii* was associated with the growth of the organisms such as lipid A biosynthesis, DNA replication or translation (Sup. Fig 3), which indicates activation of their metabolism.

Moreover, iron transport was also altered in both organisms in the presence of cortisol (Sup. Fig 3). These results are in agreement with the effects observed for the whole oral microbiome where members of the Fusobacteriales order are more active when cortisol was added to the medium, (Fig. 1). Likewise, the intersection of common molecular functions enriched with the addition of cortisol in *F. nucleatum* and *L. goodfellowii* include iron acquisition (iron-ion binding and iron-ion transmembrane transporter activity) and peptidase activities (serine-type endopeptidase activity) (Sup. Fig. 4).

Altogether, these results show that the only exposure to cortisol in the oral microbiome is enough to cause a significant shift in the gene expression profile of the community mimicking community-wide expression profiles observed in periodontitis and its progression in vivo.^22,23^ Our results also highlight the importance of *Fusobacteria* and *Leptotrichia* as the members of the community that more rapidly increase their metabolism in response to an increase of cortisol in the environment.

Though it has been known for a long time that hormones had some effect on the metabolism of bacteria,^7,25–27^ the mechanisms by which this cross-talk happens to remain mostly unknown. As we deepen our understanding of the precise roles of the microbiome in health and disease, we expect that new mechanisms will be shown to involve host hormones, including novel interactions. The present manuscript builds on the idea that human hormones can be used by the microbiome as signals to sense changes in their environment thus modifying its expression profile to fit the new conditions better. Nonetheless, we should recognize that given the limited number of samples in this pilot study, further work with a larger sample size should be performed to confirm the results presented in this manuscript.

Using a metatranscriptomic analysis would allow us to assess the direct effect that hormones have on the metabolism of the microbial community. Approaching the host-microbiome interactions from a microbial endocrinology-based point may provide an understanding of the specific pathways by which microorganisms may influence the outcome of certain infections and chronic diseases.

## Methods

All aspects of the study protocol were approved by the Institutional Review Board at The Forsyth Institute. Given the nature of the study ethical approval and the participant’s written informed consent was waived. A detailed description of the methods and bioinformatic analysis are provided in the Supplementary Material section.

## Electronic supplementary material

Supplemental material

## Data Availability

The sequence datasets used in these analyses were deposited at the Human Oral Microbiome Database (HOMD) under the submission number 20180522 (ftp://homd.org/publication_data/20180522/).

## Electronic supplementary material

**Supplementary information** accompanies the paper on the *npj Biofilms and Microbiomes* website (10.1038/s41522-018-0068-z).

## References

[1] Human Microbiome Project Consortium. (2012). Structure, function and diversity of the healthy human microbiome. Nature.

[2] McCray CJ, Agarwal SK (2011). Stress and autoimmunity. Immunol. Allergy Clin. North Am..

[3] Marcovecchio ML, Chiarelli F (2012). The effects of acute and chronic stress on diabetes control. Sci. Signal..

[4] Akcali A, Huck O, Tenenbaum H, Davideau JL, Buduneli N (2013). Periodontal diseases and stress: a brief review. J. Oral Rehabil..

[5] Gur TL (2015). Stress and the commensal microbiota: importance in parturition and infant neurodevelopment.. Front. Psychiatry.

[6] Freestone P (2013). Communication between bacteria and their hosts. Scientifica.

[7] Evans DG, Miles AA, Niven JSF (1948). The enhancement of bacterial infections by adrenaline. Br. J. Exp. Pathol..

[8] Lyte M (2013). Microbial endocrinology in the microbiome-gut-brain axis: how bacterial production and utilization of neurochemicals influence behavior. PLoS Pathog.

[9] Freestone PPE, Sandrini SM, Haigh RD, Lyte M (2008). Microbial endocrinology: how stress influences susceptibility to infection. Trends Microbiol..

[10] Mudd AT, Berding K, Wang M, Donovan SM, Dilger RN (2017). Serum cortisol mediates the relationship between fecal Ruminococcus and brain N-acetylaspartate in the young pig. Gut Microbes.

[11] Jentsch HFR, März D, Krüger M (2013). The effects of stress hormones on growth of selected periodontitis related bacteria. Anaerobe.

[12] Roberts A (2002). Stress and the periodontal diseases: effects of catecholamines on the growth of periodontal bacteria in vitro. Oral Microbiol. Immunol..

[13] Genco RJ (1998). Models to evaluate the role of stress in periodontal disease. Ann. Periodontol..

[14] Rai B, Kaur J, Anand SC, Jacobs R (2011). Salivary stress markers, stress, and periodontitis: a pilot study. J. Periodontol..

[15] Ishisaka A (2008). Association of cortisol and dehydroepiandrosterone sulphate levels in serum with periodontal status in older Japanese adults. J. Clin. Periodontol..

[16] Cakmak O, Tasdemir Z, Aral CA, Dundar S, Koca HB (2016). Gingival crevicular fluid and saliva stress hormone levels in patients with chronic and aggressive periodontitis. J. Clin. Periodontol..

[17] Davis MPA, van Dongen S, Abreu-Goodger C, Bartonicek N, Enright AJ (2013). Kraken: a set of tools for quality control and analysis of high-throughput sequence data. Methods.

[18] Segata N (2011). Metagenomic biomarker discovery and explanation. Genome Biol..

[19] Han YW (2015). Fusobacterium nucleatum: a commensal-turned pathogen. Curr. Opin. Microbiol..

[20] Park OJ (2015). Pyrosequencing analysis of subgingival microbiota in distinct periodontal conditions. J. Dent. Res..

[21] Huang S (2011). Preliminary characterization of the oral microbiota of Chinese adults with and without gingivitis. BMC Oral Health.

[22] Duran-Pinedo AE (2014). Community-wide transcriptome of the oral microbiome in subjects with and without periodontitis. ISMEJ.

[23] Yost S, Duran-Pinedo AE, Teles R, Krishnan K, Frias-Lopez J (2015). Functional signatures of oral dysbiosis during periodontitis progression revealed by microbial metatranscriptome analysis. Genome Med..

[24] Yost S, Duran-Pinedo AE, Krishnan K, Frias-Lopez J (2017). Potassium is a key signal in host-microbiome dysbiosis in periodontitis. PLOS Pathog..

[25] Sandrini S, Alghofaili F, Freestone P, Yesilkaya H (2014). Host stress hormone norepinephrine stimulates pneumococcal growth, biofilm formation and virulence gene expression. BMC Microbiol.

[26] Calil CM (2014). Effects of stress hormones on the production of volatile sulfur compounds by periodontopathogenic bacteria. Braz. Oral Res.

[27] Lyte M (2004). Microbial endocrinology and infectious disease in the 21st century. Trends Microbiol..

