# Supplementary material for: The effect of the stress hormone cortisol on the metatranscriptome of the oral microbiome
Source: NPJ Biofilms Microbiomes. 2018 Oct 18;4:25. doi: 10.1038/s41522-018-0068-z (PMC6194028; doi:10.1038/s41522-018-0068-z)
Supplement: Supplementary file 1 — Supplemental material [file 41522_2018_68_MOESM1_ESM.pdf]

Supplementary methods

Challenging the oral microbiome with cortisol

To assess the effect that cortisol has on the oral microbiome we performed metatranscriptome analysis of a biological triplicate of subgingival dental plaque from a periodontally healthy subject. The periodontal status of the donor was evaluated at the Forsyth Institute Clinic according to criteria described by the American Academy of Periodontology[1] (presented ≤ 10% of sites with BOP, no PD or CAL > 3 mm). Samples were taken separately from six individual molar sites with no BOP and PD or CAL < 3 mm using sterile Gracey curettes. Plaque samples and saliva came from the same subject pooled and were resuspended in 6 ml of saliva. The saliva+plaque suspension was mixed by gentle vortexing and aliquoted in 1ml volume per well in a 24-well Corning™ Costar™ Flat Bottom Cell Culture Plate (Thermo Fisher).

To three of those wells, we added hydrocortisone-water soluble (Sigma-Aldrich) to a final cortisol concentration of 3.5µg/ml (10µl per well of a dilution 1/100 from a stock solution of 35mg/ml), while we did not add anything to the other three wells that were used as controls. The plate was incubated at 37ºC for 2 hours under anaerobic conditions. Cells were collected by centrifugation at 10,000 x g for 5 minutes, and RNA was extracted immediately for further analysis as described below.

Effect of cortisol on *Leptotrichia goodfellowii* and *Fusobacterium nucleatum*

We also challenged *L. goodfellowii* and *F. nucleatum* with cortisol to assess the effect that this hormone has on their expression profiles. Both organisms were grown on 10mL of BD BBL Schaedler broth (Thermo Fisher) at 37ºC under anaerobic conditions until reaching an OD_{600} = 0.5. Cells were collected by centrifugation at 10,000 x g for 5 minutes, washed and resuspended in 10 ml of modified saliva medium (MSM) as described by Pratten et al. [2]. We used artificial saliva medium to avoid variability among actual saliva samples from volunteers that could influence the results. MSM composition was: yeast extract 2 g/l (BD Biosciences), proteose peptone 5 g/l (Sigma-Aldrich), porcine gastric mucin 2.5 g/l (Sigma-Aldrich), sodium chloride 0.2 g/l, potassium chloride 0.2 g/l (Sigma-Aldrich), calcium chloride 0.3 g/l; 1.25 ml/l of a 0.2 µm filter-sterilized solution of 40% urea

was added after autoclaving. MSM medium was allowed to stay at least 60 h under anaerobic conditions before inoculation.

1ml of the suspension per well was added to a 24-well Corning™ Costar™ Flat Bottom Cell Culture Plate (Thermo Fisher). To three of those wells, we added hydrocortisone/cortisol (Sigma-Aldrich) to a final cortisol concentration of 3.5μg/ml while three wells with the suspension of the microorganisms but without cortisol were used as controls. The plate was incubated at 37°C for 2 hours under anaerobic conditions. Cells were collected by centrifugation at 10,000 x g for 5 minutes, and RNA was extracted immediately for further analysis as described below. In the case of these pure cultures, RNA was not amplified for analysis. The rest of the procedure is identical to the one used for metatranscriptomic analysis of the whole community.

Metatranscriptomic analysis.

Detailed protocols for community RNA extraction, RNA amplification, and Illumina Sequencing are described in Yost et al. [3]. Briefly, 600μL of mirVana kit lysis/binding buffer and 300 μl of 0.1-mm zirconia-silica beads (BioSpec Products) were added to the samples. Samples were bead beaten for 1 min at maximum speed. RNA was extracted following the protocol of *the mir*Vana™ Isolation kit for RNA. MICROB*Express* (Life Technologies) was used to remove prokaryotic rRNA. All kits were used following the manufacturer's instructions. RNA amplification was performed on total bacterial RNA using MessageAmp™ II-Bacteria RNA amplification kit (Life Technologies) following the manufacturer's instructions. Sequencing was performed at the Forsyth Institute.

For the bioinformatic analysis, we first calculated the biological variation (BCV) after estimating the common dispersion using the R package edgeR. These values were used as a cv (coefficient of variation) cutoff in NOISeq. For the whole community, analysis cv was 2 (200 cutoff NOISeq), for the *Leptotrichia goodfellowii* libraries cv was 1.2 (120 cutoff NOISeq) and for the *Fusobacterium nucleatum* libraries cv was 0.28 (28 cutoff NOISeq).

Low-quality sequences were removed from the query files. Fast clipper and fastq quality filter from the Fastx-toolkit (http://hannonlab.cshl.edu/fastx toolkit/) were used to remove short sequences with a quality score >20 in >80% of the sequence. Cleaned files were then aligned against the bacterial/archaeal database using bowtie2. We generated a .gff file to map hits to different regions in

the genomes of our database. Read counts from the SAM files were obtained using bedtools multicov from bedtools [4].

To identify deferentially expressed (DE) genes from the RNA libraries, we applied non-parametric tests to the normalized counts using the NOISeqBio function of the R package 'NOISeq' with 'tmm' normalization, with batch and length correction and removing genes whose sum of hits across samples was lower than 10. We used a significance threshold value of q=0.95, which is equivalent to an FDR adjusted p-value of 0.05[5].

To evaluate functional activities deferentially represented we mapped the DE genes to Gene Ontology (GO) terms (http://www.geneontology.org/). GO terms for the different ORFs were obtained from the PATRIC database (http://patricbrc.org/portal/portal/patric/Home). GO terms not present in the PATRIC database and whose annotation was obtained from the HOMD database or the J. Craig Venter Institute were acquired using the program blast2GO under the default settings [6]. Enrichment analysis on these sets was performed using the R package 'GOseq,' which accounts for biases due to over-detection of long and highly expressed transcripts [6]. Gene sets with ≤ ten genes were excluded from analysis. We used the REVIGO web page [7] to summarize and remove redundant GO terms. Only GO terms with FDR adjusted p-value < 0.05 in the 'GOseq' analysis were used.

Phylogenetic assignment of transcripts (LEfSe)

Counts from the mRNA libraries were used to determine their phylogenetic composition for bacteria and archaea. Phylogenetic profiles of the metatranscriptomes were obtained using Kraken [8]. We generated a custom Kraken library with the oral microbiome genomes indicated in the above section with a filtering threshold of 0.05. Phylogenetic profiles were used to identify significant differences between active communities under the different conditions studied by performing linear discriminant analysis (LDA) effect size (LEfSe) as proposed by Segata et al. [9] with an alpha value for the Wilcoxon test to 0.01. Significant *P*-values associated with microbial clades and functions identified by LEfSe were corrected for multiple hypothesis testing using the Benjamini and Hochberg false discovery rate correction [10] using the p.adjust function in R with a cutoff of FDR < 0.05.

Methods References

1. Armitage, G. C. Development of a classification system for periodontal diseases and conditions. *Ann. Periodontol.* **4,** 1–6 (1999).
2. Pratten, J., Wills, K., Barnett, P. & Wilson, M. In vitro studies of the effect of antiseptic-containing mouthwashes on the formation and viability of Streptococcus sanguis biofilms. *J. Appl. Microbiol.* **84,** 1149–1155 (1998).
3. Yost, S., Duran-Pinedo, A. E., Teles, R., Krishnan, K. & Frias-Lopez, J. Functional signatures of oral dysbiosis during periodontitis progression revealed by microbial metatranscriptome analysis. *Genome Med* **7,** 27 (2015).
4. Quinlan, A. R. & Hall, I. M. BEDTools: a flexible suite of utilities for comparing genomic features. *Bioinformatics* **26,** 841–842 (2010).
5. Tarazona, S., García-Alcalde, F., Dopazo, J., Ferrer, A. & Conesa, A. Differential expression in RNA-seq: a matter of depth. *Genome Res.* **21,** 2213–2223 (2011).
6. Young, M. D., Wakefield, M. J., Smyth, G. K. & Oshlack, A. Gene ontology analysis for RNA-seq: accounting for selection bias. *Genome Biol* **11,** R14 (2010).
7. Supek, F., Bošnjak, M., Škunca, N. & Šmuc, T. REVIGO summarizes and visualizes long lists of gene ontology terms. *PLoS ONE* **6,** e21800 (2011).
8. Davis, M. P. A., van Dongen, S., Abreu-Goodger, C., Bartonicek, N. & Enright, A. J. Kraken: a set of tools for quality control and analysis of high-throughput sequence data. *Methods* **63,** 41–49 (2013).
9. Segata, N. *et al.* Metagenomic biomarker discovery and explanation. *Genome Biol* **12,** R60 (2011).
10. Benjamini, Y. & Hochberg, Y. Controlling the false discovery rate: a practical and powerful approach to multiple testing. *J. Roy. Statist. Soc. Ser. B* **57,** 289–300 (1995).

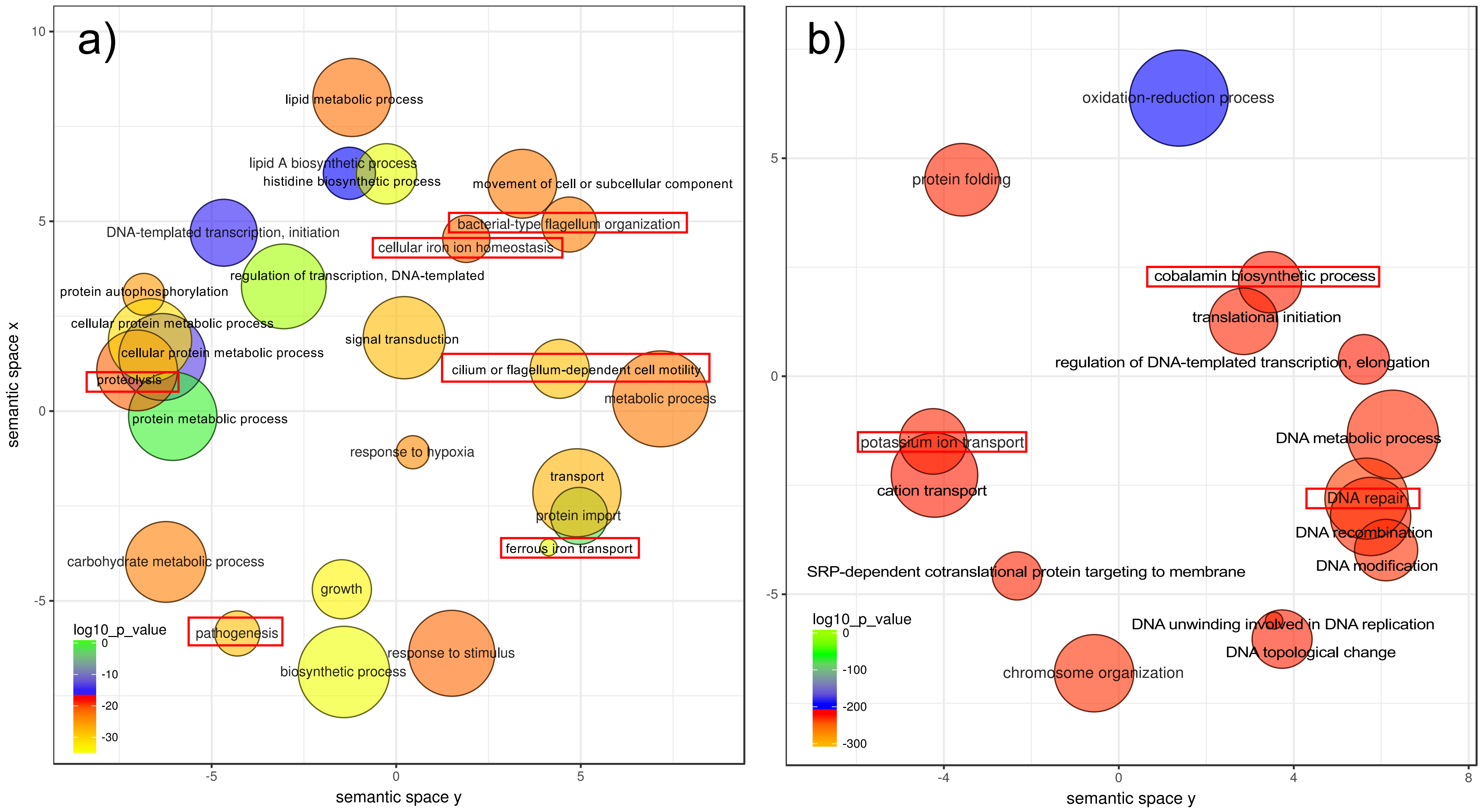

Figure S1. Community-wide GO enrichment analysis of diferentially expressed putative virulence factors in response to the presence and absence of added cortisol to the medium. Putative virulence factors were identified by alignment of the protein sequences from the different genomes against the Virulence Factors Database (VFDB) as described in the methods section. Enriched terms obtained using GOseq were summarized and visualized as a scatter plot using REVIGO. Only GO terms with FDR adjusted p-value < 0.05 in the 'GOseq' analysis were used.
A) Summarized GO terms related to biological processes after addition of cortisol. B) Summarized GO terms related to biological processes with no cortisol added.
Circle size is proportional to the frequency of the GO terms; color indicates the log10 p-value (red higher, blue lower). The distance between circles represents GO terms' semantic similarities.
Each of the circles represents a GO term, which depending on the similarity in the terms included in them they will be closer or more distant in the graph.
In red are activities we have previously seen associated with periodontitis (19, 20).

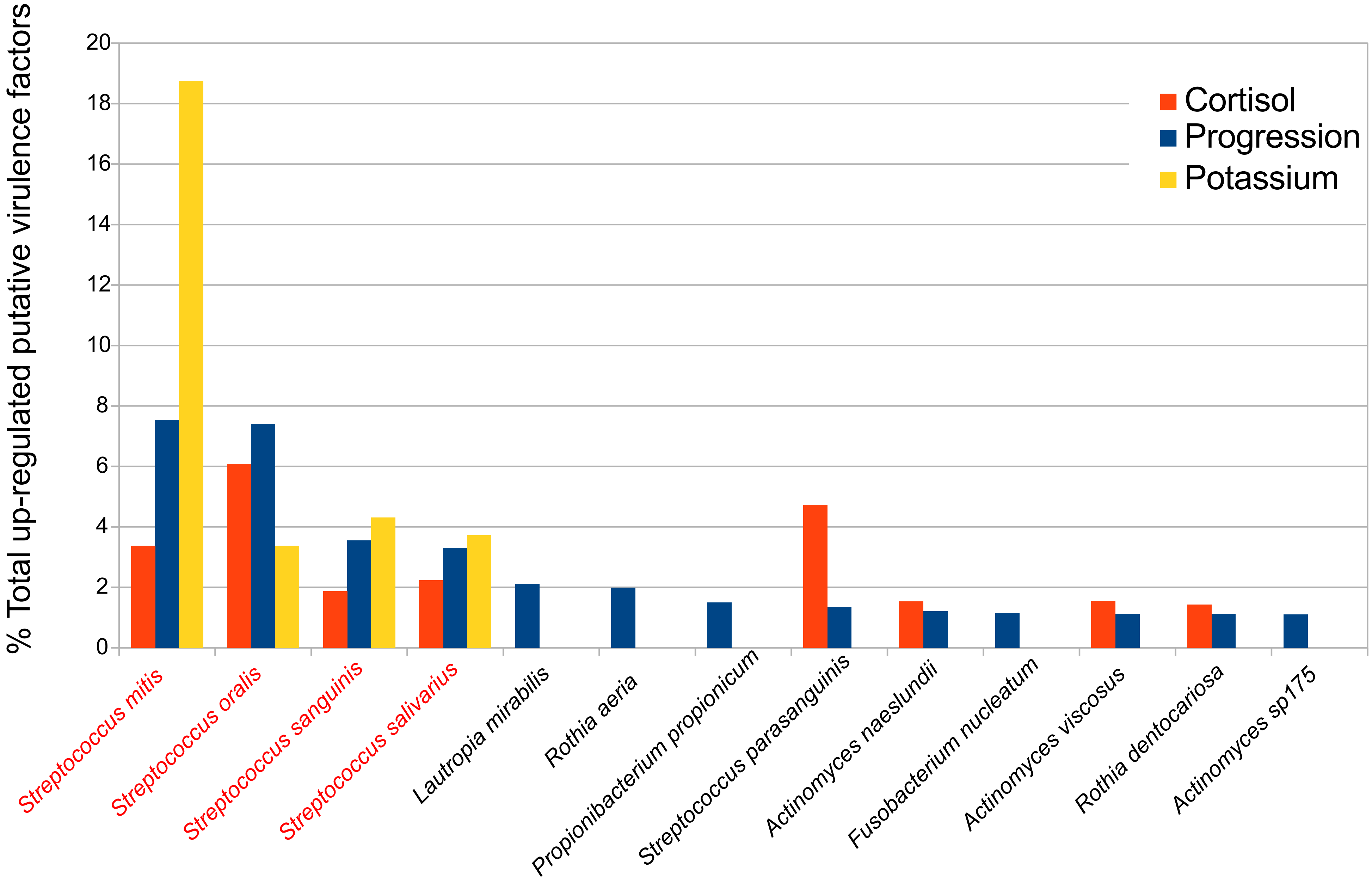

Figure S2.Species up-regulating putative virulence factors in the presence of cortisol. We ranked the species based on the number of up-regulated putative virulence factors observed in the metatranscriptome of the oral microbiome. Putative virulence factors were identified by alignment of the protein sequences from the different genomes against the Virulence Factors Database (VFDB) as described in the methods section. Numbers in the graph refer to the percentage of hits of the different species for the putative virulence factors identified. We selected only species whose percentage of putative virulence factors from the total of the community was higher than 1%.
We included also results from 2 previous studies one on periodontal disease progression [21] and another where we showed that potassium was a crucial signal in dysbiosis [22].
In red, species that were ranked at the top of putative virulence factors up-regulation in all three studies**.**

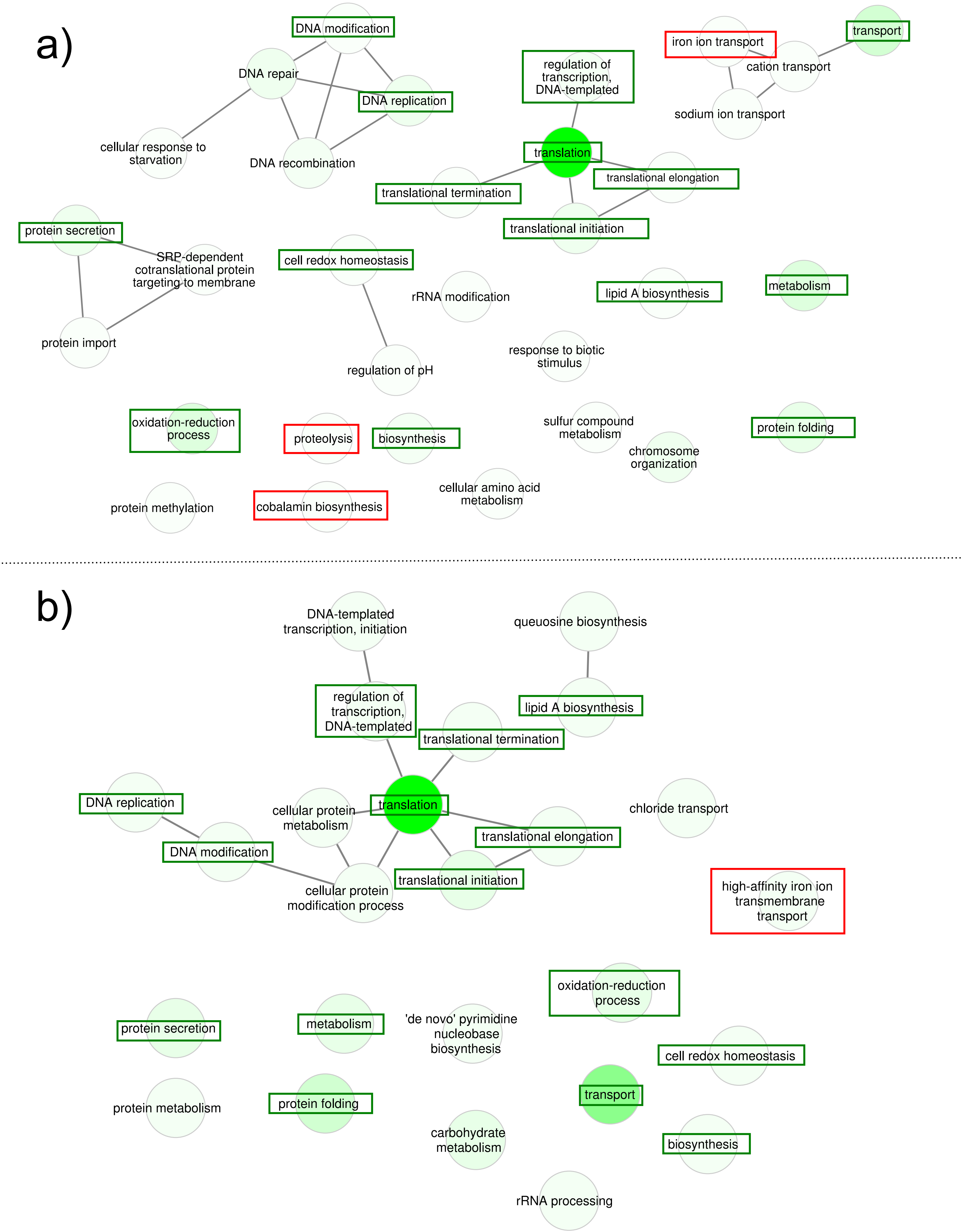

Figure S3. Changes in functional profiles in pure cultures of Fusobacterium nucleatum and Leptotrichia goodfellowii. We used Gene Ontology (GO) enrichment analysis to assess the functional response of *Fusobacterium nucleatum* and *Leptotrichia goodfellowii* to the presence of added cortisol to the medium. Biological processes GO terms associated with changes in gene expression profiles in *F. nucleatum* and *L. goodfellowii*.
GO terms were assigned to diferentially expressed genes due to the addition of cortisol and summarized using REVIGO.
a) GO terms associated with up-regulated genes in *F. nucleatum* b) GO terms associated with down-regulated genes *L. goodfellowi*i.
In green are metabolic activities that were associated with up-regulated genes in both F. nucleatum and L. goodfellowii.
In red are activities we have previously seen associated with periodontitis [20,21].

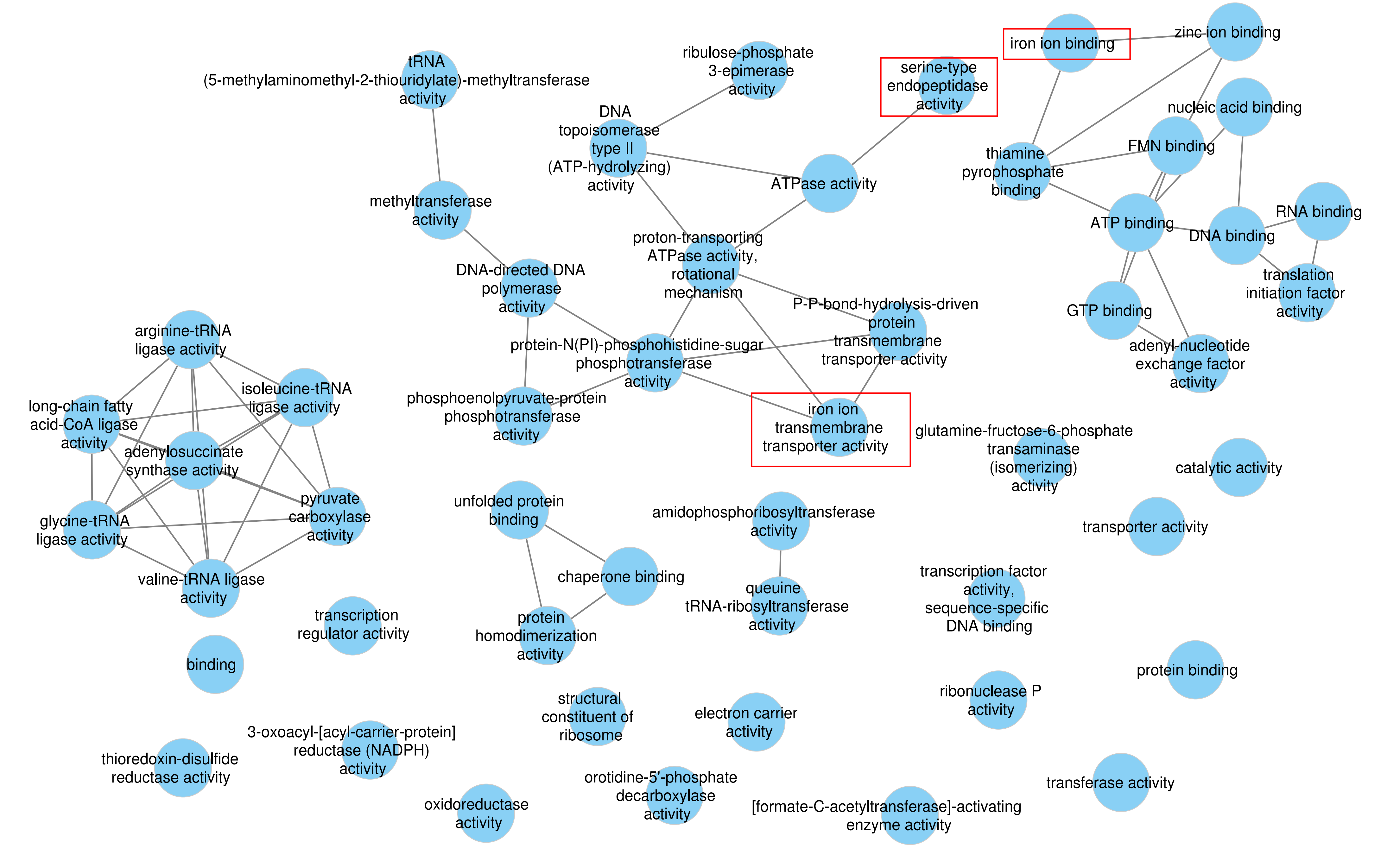

Figure S4. Common molecular function GO terms associated with changes in gene expression profiles in *Fusobacterium nucleatum and Leptotrichia goodfellowii*.
Gene Ontology (GO) terms were assigned to diferentially expressed genes due to the addition of cortisol and summarized using REVIGO. Networks of over-represented molecular functions from *F. nucleatum* and *L. goodfellowii* were then uploaded to Cytoscape, and the intersection of the two networks was extracted and plotted as shown in the figure.
In red are activities we have previously seen associated with periodontitis [20,22].
